# The Treatment of Eclampsia

**Published:** 1902-04-05

**Authors:** 


### THE TREATMENT OF ECLAMPSIA.

At the last meeting of the Medical Society Dr. Herman, in opening a discussion on the treatment of puerperal eclampsia, raised the questions whether emptying the uterus was the appropriate treatment, and whether it would stop the fits. He showed that the teaching of both old and recent writers was to the effect that emptying the uterus stopped the fits, and that this proceeding should, therefore, be the first thing in treatment. He pointed out, however, that although this had been taught for at least three generations it still needed proof, and he brought statistics, drawn from the practice of many lying-in hospitals, showing that in every institution in which experience was large enough it was found that the fits continued after delivery about as often as they ceased with it. Thus the general statement that "after delivery the fits at once cease" could not be maintained. Entering then on the further question whether, putting on one side the cessation of the fits, the acceleration of delivery might not favour recovery, he showed that in maternities conducted with strict asepsis the mortality among eclampsia cases which were delivered natuıally was about the same as among those in which the emptying of the uterus was accelerated, while in pre-antiseptic days the accelerated cases had been in some institutions much more fatal than those delivered naturally. As a prophylactic, however, he fully believed in the propriety of inducing premature labour when there was much albumin in the urine. Commenting on this interesting paper Dr. G. F. Blacher also questioned the advantage of interfering locally. He thought there were three indications: first to lessen the reflex irritability of the nervous system; second, to eliminate the poison by free venesection; and third, to dilute the poison which remained by intravenous injection of considerable doses of saline solution.

